# The RASSF1 Gene and the Opposing Effects of the RASSF1A and RASSF1C Isoforms on Cell Proliferation and Apoptosis

**DOI:** 10.1155/2013/145096

**Published:** 2013-11-12

**Authors:** Mark E. Reeves, Matthew Firek, Shin-Tai Chen, Yousef Amaar

**Affiliations:** ^1^Surgical Oncology Laboratory, Loma Linda VA Medical Center, Loma Linda, CA 92357, USA; ^2^Department of Surgery, Loma Linda University, Loma Linda, CA 92357, USA; ^3^Musculoskeletal Disease Center, Loma Linda VA Medical Center, Loma Linda, CA 92357, USA

## Abstract

RASSF1A has been demonstrated to be a tumor suppressor, while RASSF1C is now emerging as a growth promoting protein in breast and lung cancer cells. To further highlight the dual functionality of the RASSF1 gene, we have compared the effects of RASSF1A and RASSF1C on cell proliferation and apoptosis in the presence of TNF-**α**. Overexpression of RASSF1C in breast and lung cancer cells reduced the effects of TNF-**α** on cell proliferation, apoptosis, and MST1/2 phosphorylation, while overexpression of RASSF1A had the opposite effect. We also assessed the expression of RASSF1A and RASSF1C in breast and lung tumor and matched normal tissues. We found that RASSF1A mRNA levels are significantly higher than RASSF1C mRNA levels in all normal breast and lung tissues examined. In addition, RASSF1A expression is significantly downregulated in 92% of breast tumors and in 53% of lung tumors. Conversely, RASSF1C was upregulated in 62% of breast tumors and in 47% of lung tumors. Together, these findings suggest that RASSF1C, unlike RASSF1A, is not a tumor suppressor but instead may play a role in stimulating survival in breast and lung cancer cells.

## 1. Introduction

RASSF1A and RASSF1C are the two major isoforms encoded by the RASSF1 gene. RASSF1A is a key tumor suppressor. It is inactivated in the vast majority of human cancers, including breast and lung cancers [1–5]. Recently, we and others demonstrated that the RASSF1C isoform promotes cell proliferation and migration and attenuates apoptosis in breast and lung cancers [6–10]. This suggests that RASSF1C and RASSF1A have opposing effects on cancer cells. This is further supported by recent cohort studies in human lung and pancreatic cancers showing that elevated RASSF1C expression correlates significantly with poor patient survival [11, 12].

To shed more light on this newly emerging concept of RASSF1 dual functionality, we have undertaken studies which compare the impact of the RASSF1A and RASSF1C isoforms on TNF-*α*-induced apoptosis. RASSF1A is intimately involved in the Hippo and MOAP1/Bax proapoptotic pathways. RASSF1A promotes apoptosis through activation of both MST kinases in the Hippo pathway and activation of Bax in the MOAP/Bax pathway through TNF-*α* receptor activation. RASSF1A promotes and maintains phosphorylation of MST1, and also activates Bax leading to the induction/activation of downstream of proapoptotic genes such as puma and caspases 3 and 7 genes [13–17]. Like RASSF1A, RASSF1C is able to bind MST1/2 proteins. As such, we reasoned that RASSF1C could potentially interfere with MST1/2 activation and antagonize the effects of RASSF1A. Therefore, we used the TNF-*α*-induced apoptosis system to compare and contrast the effects of RASSF1A and RASSF1C in breast and lung cancer cells. To do this, we overexpressed RASSF1A and RASSF1C in both breast and lung cancer cells. We then used these cells to first assess cell proliferation and then to measure caspase 3/7 activation and MST phosphorylation in the presence of TNF-*α*. Lastly, we also assessed the expression of RASSF1A and RASSF1C in breast and lung tumor and normal tissues.

## 2. Materials and Methods

### 2.1. Cell Culture

The human breast cancer cell line T47D and lung cancer cell line NCI-H1299 stably overexpressing HA-RASSF1A or HA-RASSF1C were developed as previously described [6]. Cells overexpressing HA-RASSF1A (T47D-1A, H1299-1A), HA-RASSF1C (T47D-1C, H1299-1C), or the empty MLV backbone (T47D-BB, H1299-BB) were grown in RPMI-1640 medium supplemented with 10% calf bovine serum. For growth of T47D cells, medium was supplemented with 0.2 U/mL insulin.

### 2.2. Western Blot Analysis

Western blot analysis was carried out using standard procedures with detection on the Odyssey Infrared System (LI-COR Biosciences, Lincoln, NE). Rabbit polyclonal anti-MST1/2 and anti-pMST1/2 (Cell Signaling), HA-tag antibody (Covance), anti-YAP and anti-pYAP (Cell Signaling), and goat anti-mouse and donkey anti-rabbit fluorescently labeled secondary antibodies (IRDye 680 926-32220, LI-COR Biosciences) were used.

### 2.3. Treatment of Cells with TNF-*α*

Cells were plated in 96-well plates at 5000 cells/well and were treated the next day with 1 *μ*g/mL Actinomycin D for 2 hr. Cells were then treated with 40 *μ*M TNF-*α* in DMSO (Sigma, St. Louis, MO) for 6 hr and then analyzed by western blot. Alamar Blue cell viability assays were performed 18 hr after TNF-*α* treatment as previously described [7]. Cell proliferation was measured by the Alamar Blue assay as previously described [6, 7]. Data are presented as mean values ± SEM and analyzed with Student's *t*-test. Values ≤0.05 were considered significant.

### 2.4. Caspase 3/7 Activity Assay

Cells treated with TNF-*α* for 6 hr were assayed for caspase 3/7 activity using the Apo3/7 caspase activity assay (Promega, Madison, WI).

### 2.5. RNA Isolation and RT-PCR Analysis

Tumor and matched normal tissues were obtained from the Western Division of the Cooperative Human Tissue Bank (Vanderbilt University, TN).

Tumor and normal breast and lung tissues were ground to a powder using a mortar and pestle, and the tissue powder was used to isolate total RNA using PureLink RNA Mini Kit (Invitrogen, Carlsbad, CA). 1 *μ*g of total RNA was used in reverse transcriptase (RT) reactions using the superscript kit (Qiagen, Germantown, MD) and the RT reactions were subsequently used to set up real-time PCR (RT-PCR) reactions using 1 *μ*L of RT as a template. The RT-PCR reactions were set up using SYBR green PCR master mix (BioRad, Hercules, CA) and the PCR reactions were run using the Opticon 2 PCR machine (BioRad). Cyclophilin was used as a loading control. Expression of RASSF1A and RASSF1C was assessed by qRT-PCR using RASSF1A and RASSF1C specific primers. The PCR reactions were run using the following protocol: (1) incubate at 95°C for 10 min, (2) incubate at 95°C for 15 sec, (3) incubate for 30 sec, (4) go to line 2 for 39 more cycles, (5) melt curve from 60°C to 95°C, read every 1.0°C, and (6) incubate at 10°C forever. qRT-PCR reactions were carried out in triplicate and the fold change was calculated using the 2^−ΔΔCT^ method [18]. The RASSF1A forward primer is 5′ GGCGTCGTGCGCAAAGGCC 3′, the RASFF1C forward primer is 5′ CTGCAGCCAAGAGGACTCGG 3′, and the reverse primer for both RASSF1A and RASSF1C is 5′ GGGTGGCTTCTTGCTGGAGGG 3′. The cyclophilin (housekeeping gene) forward primer is 5′ GCATACAGGTCCTGGCATCT 3′, and the cyclophilin reverse primer is 5′ TCTTGCTGGTCTTGCCATTC 3′. PCR reactions were set in triplicates and cyclophilin was used as an internal loading control and was used to normalize the relative expression levels using the 2^−ΔΔCT^ method [18].

## 3. Results

### 3.1. RASSF1C Reduces the Effects of TNF-*α* on Cell Proliferation

**Figure 1:**
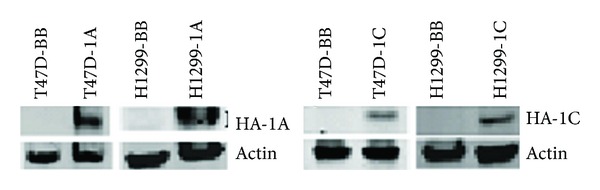
Breast T47D and lung NCI-H1299 cancer cells stably transduced with MLV backbone or MLV vectors containing HA-RASSF1A or HA-RASSF1C overexpress the transduced protein as judged by western blots using HA-antibody. T47D-BB = T47D transduced with MLV backbone, T47D-1A = T47D overexpressing HA-RASSF1A, T47D-1C = T47D overexpressing HA-RASSF1C, H1299-BB = NCI-H1299 transduced with MLV backbone, and H1299-1A = NCI-H1299 overexpressing HA-RASSF1A, H1299-1C = NCI-H1299 overexpressing HA-RASSF1C.

**Figure 2:**
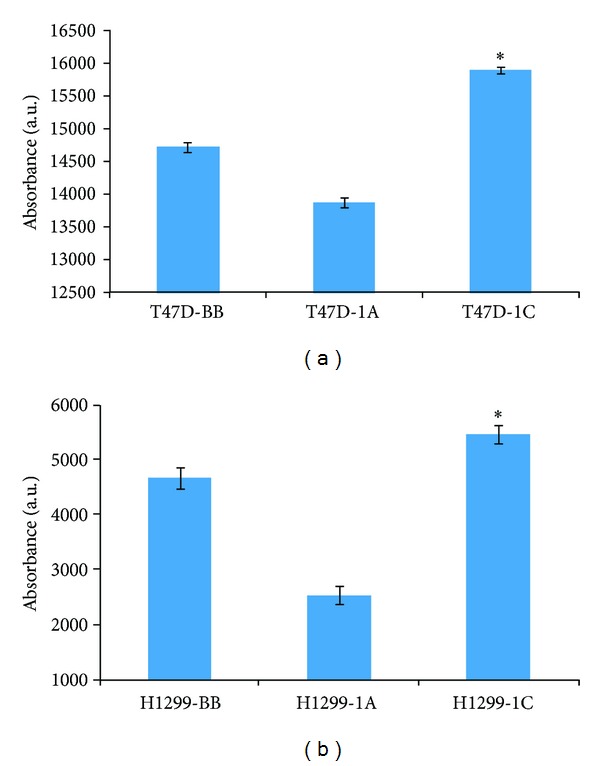
T47D breast (a) and H1299 lung (b) cancer cells stably overexpressing RASSF1A or RASSF1C were assayed for cell viability/proliferation in absence of TNF-*α* using the Alamar Blue assay. RASSF1A overexpression significantly reduced while RASSF1C overexpression significantly enhanced cell viability/proliferation compared to control (BB) cells. Data is representative of at least 3 independent experiments and the values represent the mean ± SEM. *P < 0.05 for 1C compared to 1A and BB and for 1A compared to BB.

**Figure 3:**
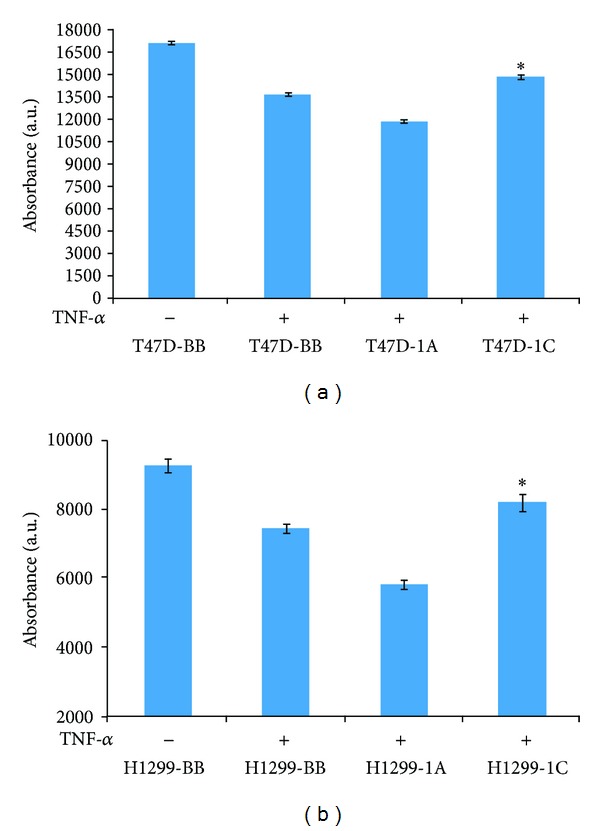
T47D breast (a) and NCI-H1299 lung (b) cancer cells overexpressing RASSF1A or RASSF1C were treated with TNF-*α* for 24 hr and then cells were assayed for cell viability/proliferation. RASSF1A overexpression significantly enhanced while RASSF1C overexpression significantly reduced the sensitivity of cells to TNF-*α*. Data is representative of at least 3 independent experiments and the values represent the mean ± SEM. *P < 0.05 for 1C compared to 1A and BB and for 1A compared to BB.

TNF-*α* induces cell apoptosis, and it has been demonstrated that RASSF1A mediates the apoptotic effects of TNF-*α*. In contrast, we have previously shown that overexpression of RASSF1C attenuates apoptosis in breast and lung cancer cells. In this study we compared the effects of RASSF1A and RASSF1C overexpression on breast and lung cancer cell proliferation in the presence and absence of TNF-*α* (Figures 1, 2, and 3). We found that while RASSF1A reduced cell proliferation and further enhanced the antiproliferative effects of TNF-*α*, RASSF1C significantly increased cell proliferation and reduced the antiproliferative effects of TNF-*α* on breast and lung cancer cells. Our findings suggest that RASSF1A and RASSF1C have antagonistic effects on cell proliferation.

### 3.2. RASSF1C Reduces Caspase 3/7 Activation in Breast and Lung Cancer Cells Treated with TNF-*α*

**Figure 4:**
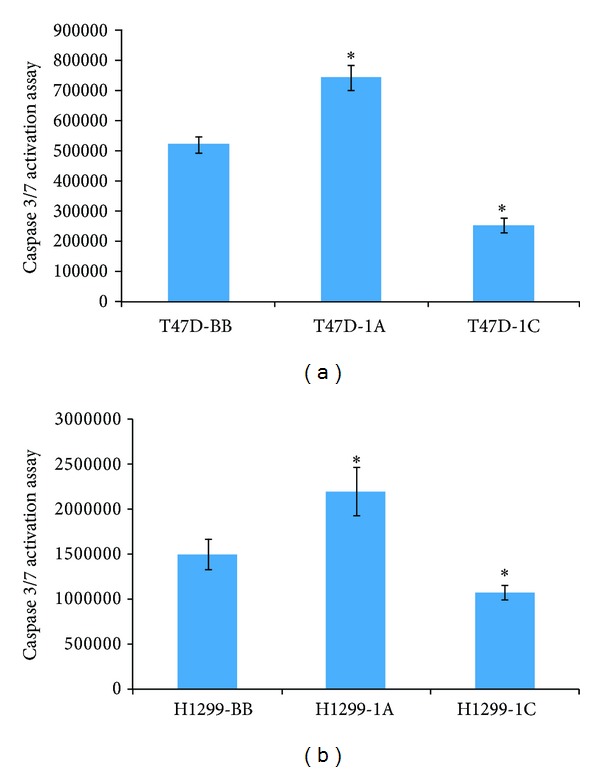
Caspase 3/7 activation was assessed in breast T47D (a) and lung H1299 (b) cancer cells overexpressing RASSF1A or RASSF1C after TNF-*α* treatment for 24 hr. Overexpression of RASSF1C significantly reduced caspase 3/7 activation in both breast and lung cancer cells. Data is representative of at least 3 independent experiments and the values represent the mean ± SEM. *P < 0.05 for 1C compared to 1A and BB and for 1A compared to BB.

To further investigate the antagonism of the antiproliferative effects of TNF-*α* by RASSF1C, we assessed the activation of caspase 3/7 in breast and lung cancer cells overexpressing RASSF1A and RASSF1C that were treated with TNF-*α*. Both breast and lung cancer cells overexpressing RASSF1C showed a significant reduction in caspase 3/7 activation, while RASSF1A overexpression enhanced caspase 3/7 activity (Figure 4). This suggests that RASSF1C has antiapoptotic effects on breast and lung cancer cells and further supports antagonistic effects of RASSF1A and RASSF1C on apoptosis.

### 3.3. RASSF1C Overexpression Effects on MST1/2 or YAP Phosphorylation

**Figure 5:**
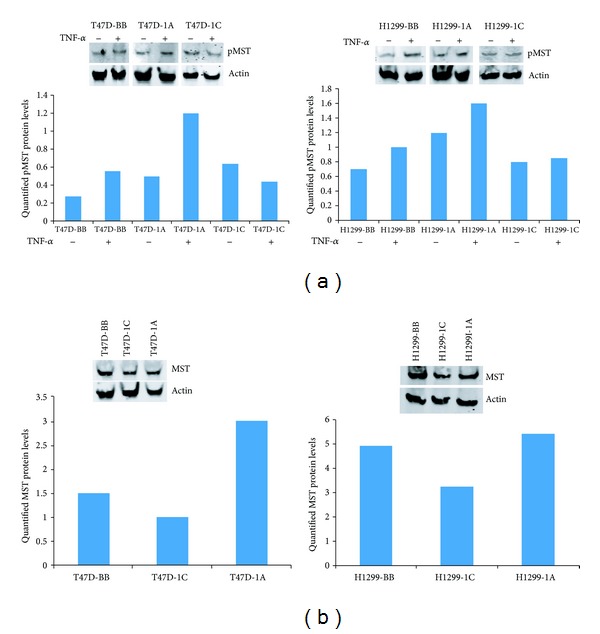
(a) These breast or lung cancer cells overexpressing RASSF1A or RASSF1C were treated with TNF-*α* and cell lysates were used to assess MST1 phosphorylation. RASSF1A overexpression increased pMST in both breast and lung cancer cells, and RASSF1C overexpression slightly reduced pMST in breast cancer cells but had no effect on pMST in lung cancer cells treated with TNF-*α*. (b) Overexpression of RASSF1C appears to reduce and RASSF1A appears to increase total MST levels compared to control. The blots shown represent one of three independent experiments.

In order to help understand how RASSF1A and RASSF1C might exhibit antagonistic effects on apoptosis, we studied the effects of overexpression of RASSF1A and RASSF1C on the Hippo pathway. While RASSF1A promotes apoptosis through the Hippo pathway by inducing mammalian sterile 20-like kinases 1 and 2 (MST1/2) phosphorylation, nothing is known about the impact of RASSF1C on MST1/2 phosphorylation or its effect on the Hippo pathway more broadly. Therefore, we assessed the phosphorylation of MST1/2 in breast and lung cancer cells overexpressing RASSF1A and RASSF1C in the presence of TNF-*α* (Figure 5). We found that in breast cancer cells, overexpression of RASSF1A enhanced MST1/2 phosphorylation as expected, but RASSF1C overexpression slightly reduced it (Figure 5(a)). However, in lung cancer cells, while RASSF1A also enhanced MST1/2 phosphorylation, overexpression of RASSF1C had no effect on MST1/2 phosphorylation (Figure 5(a)). We also assessed the levels of total MST1/2 in breast and lung cancer cells overexpressing RASSF1A or RASSF1C and found that RASSF1A appears to enhance and RASSF1C appears to reduce MST1/2 protein levels compared to control (Figure 5(b)).

**Figure 6:**
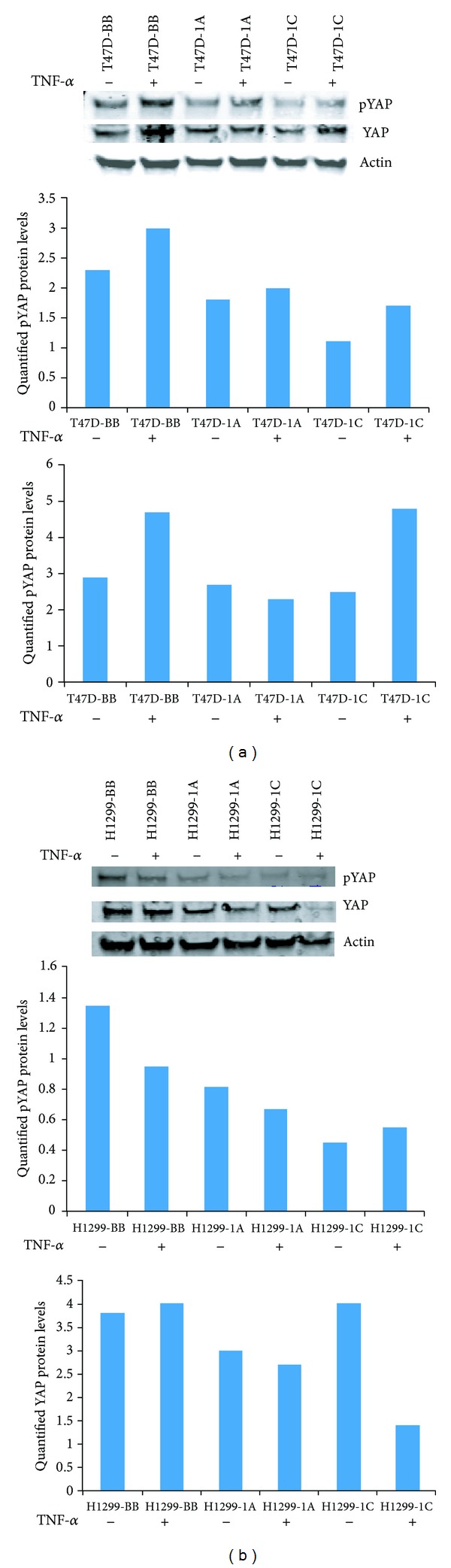
Breast T47D and lung NCI-H1299 cancer cells stably overexpressing RASSF1A or RASSF1C were treated with TNF-*α* and cell lysates were used to assess YAP phosphorylation (pYAP). (a) Breast cancer cells overexpressing RASSF1A showed a small increase in pYAP compared to cells overexpressing RASSF1C but cells overexpressing RASSF1A or RASSF1C showed reduced pYAP compared to control (BB). Overexpression of RASSF1A reduced and RASSF1C increased total YAP in the presence of TNF-*α* in breast cancer cells. (b) Overexpression of RASSF1A slightly reduced and RASSF1C overexpression slightly increased pYAP protein levels in the presence of TNF-*α* in lung cancer cells, but pYAP levels in cells overexpressing RASSF1A or RASSF1C are lower compared to those in control cells. Overexpression of RASSF1A slightly reduced and RASSF1C significantly decreased total YAP protein levels in the presence of TNF-*α* compared to those in control cells. The blots shown represent one of three independent experiments.

We also assessed the phosphorylation of the Yorkie associated protein (YAP) in the presence of TNF-*α*. YAP is a downstream effector protein in the Hippo pathway. Breast cancer cells overexpressing RASSF1A or RASSF1C exhibited an increase in phosphorylated YAP levels. However, breast cancer cells overexpressing RASSF1A had slightly decreased levels of total YAP, while cells overexpressing RASSF1C had significantly increased levels of total YAP (Figure 6(a)). In lung cancer cells, RASSF1A overexpression slightly reduced both pYAP and total YAP levels, while RASSF1C overexpression slightly increased pYAP and significantly reduced total YAP levels compared to untreated cells (Figure 6(b)). Thus, it is possible that the antiproliferative and proapoptotic effects of TNF-*α* may not involve YAP phosphorylation. 

### 3.4. RASSF1A and RASSF1C Expression in Normal Breast and Lung Tissues

**Figure 7:**
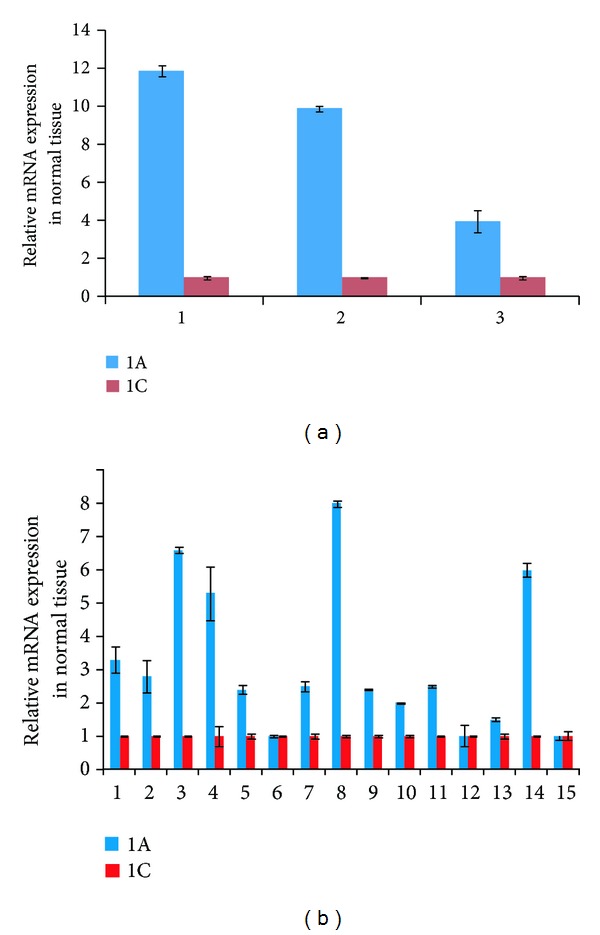
Assessment of RASSF1A and RASSF1C mRNA expression was carried out in normal breast ((a), 3 samples) and normal lung tissues ((b), 15 samples) using RT-PCR analysis. Data shows that RASSF1A mRNA levels are significantly higher than RASSF1C mRNA levels in normal breast and lung tissues. PCR reactions were set in triplicate and cyclophilin was used as an internal loading control and was used to normalize the relative expression levels using the 2^−ΔΔCT^ method [18].

The balance of the ratio of expression of RASSF1 isoforms relative to each other may be critical for maintaining normal versus transformed growth. Hence the expression of RASSF1A and RASSF1C was assessed in normal breast and lung tissues using RT-PCR (Figure 7). Interestingly, RASSF1A mRNA levels are significantly higher than RASSF1C mRNA levels in three normal breast tissues and the overwhelming majority of 15 normal lung tissues examined. This is consistent with the concept that higher RASSF1A expression levels relative to RASSF1C levels may contribute to normal cellular growth and prevent transformed cellular growth.

### 3.5. RASSF1A and RASSF1C Expression in Tumor Tissue

**Figure 8:**
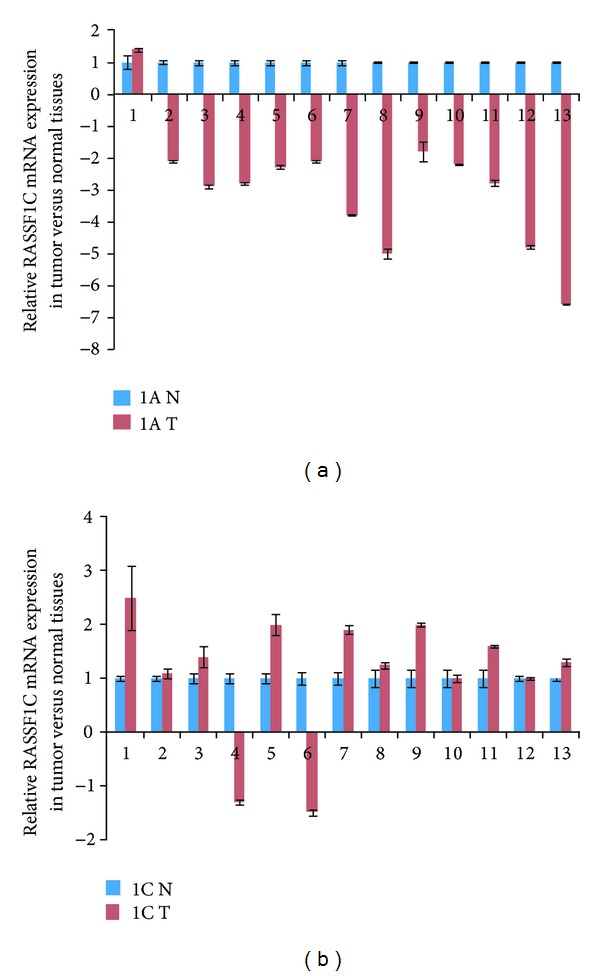
RASSF1A and RASSF1C mRNA were assessed in 13 breast tumor tissues and normal tissues using RT-PCR. (a) RASSF1A is downregulated in 92% (12/13) and is upregulated in 8% (1/13); and (b) RASSF1C is downregulated in 15% and is upregulated in 62% (8/13) of the breast tumors assessed. 1A N : relative RASSF1A expression in normal breast tissue. 1A T : relative RASSF1A expression in breast tumor tissue. 1C N : relative RASSF1C expression in normal breast tissue. 1C T : relative RASSF1C expression in breast tumor tissue.

**Figure 9:**
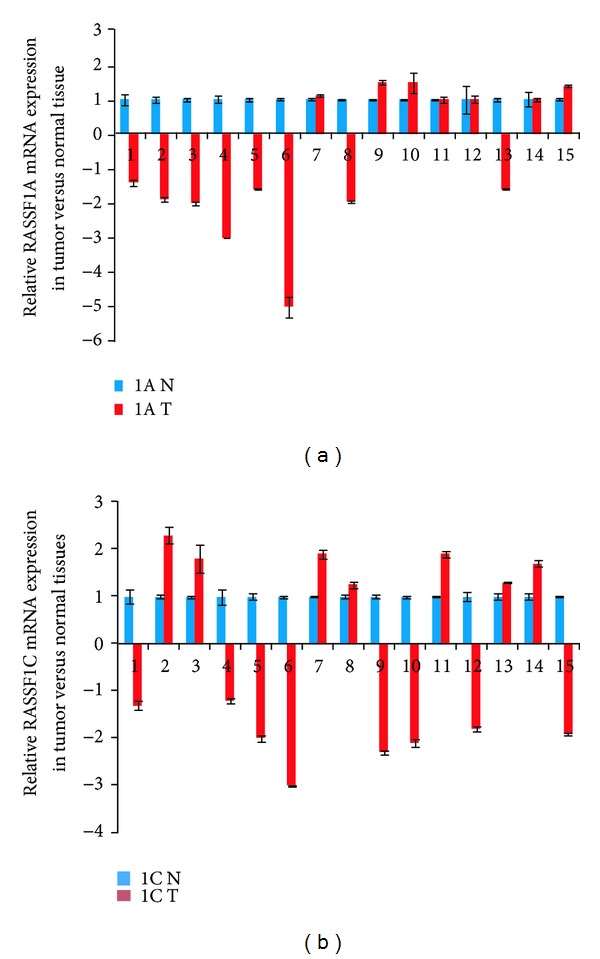
Assessment of RASSF1A and RASSF1C mRNA expression was carried out in 15 lung adenocarcinomas and matched normal tissues using RT-PCR. (a) RASSF1A was downregulated in 53% (8/15) and upregulated in 20% (3/15) and (b) RASSF1C was downregulated in 53% (8/15) and upregulated in 47% (7/15) of the lung adenocarcinomas tested.

We also assessed the expression of RASSF1A and RASSF1C mRNA levels in tumor and matched normal breast and lung tissues. Consistent with previously reported results, RASSF1A expression was significantly downregulated in 92% of breast tumors and 53% of lung adenocarcinomas (Figures 8 and 9). In contrast, RASSF1C expression was significantly upregulated in 62% of breast tumors and in 47% of lung adenocarcinomas (Figures 8 and 9). This further suggests that, unlike RASSF1A, RASSF1C is not a tumor suppressor. Rather, RASSF1C may actually have a growth promoting function in tumors. Consistent with this, we found that the ratio of RASSF1C to RASSF1A is greater than 1 in 100% of breast and 60% of lung tumors (Figure 9). Thus, it is possible that the expression ratio of RASSF1 isoforms is important for supporting normal versus oncogenic growth behavior.

## 4. Discussion

Evidence is accumulating to support the hypothesis that the RASSF1 gene may function to balance critical cellular functions that can tip the balance between normal and neoplastic behavior depending on whether the RASSF1A or RASSF1C isoform is predominantly expressed [6–12]. RASSF1A functions as a tumor suppressor by inhibiting cell proliferation and migration and promoting apoptosis [4, 5, 13]. In contrast, we and others have shown that RASSF1C has an opposite effect, promoting cancer cell proliferation and migration and attenuating apoptosis in breast and lung cancer cells [6–10]. RASSF1A mediates the apoptotic effects of TNF-*α* through the activation of modulator of apoptosis protein 1 (MOAP1) and Bax leading to caspase 3 activation [13]. TNF-*α* is an inflammatory cytokine that regulates cell proliferation and cell death, and resistance to TNF-*α*-induced apoptosis may lead to tumor formation and growth. In this study we have conducted studies directly comparing the effects of RASSF1A and RASSF1C on breast and lung cancer cell proliferation and apoptosis in the presence and absence of TNF-*α*. We confirmed that overexpression of RASSF1A reduced while overexpression of RASSF1C enhanced breast and lung cancer proliferation/viability (Figure 2). More importantly, our data show that RASSF1C reduced while RASSF1A enhanced the antiproliferative effects of TNF-*α* on breast and lung cancer cells. This adds further evidence for growth-promoting and apoptosis-attenuating functions for RASSF1C. Our current findings are consistent with our previously reported work that RASSF1C reduces the sensitivity of breast cancer cells to the chemotherapeutic agent, etoposide [8]. More recent studies show that RASSF1A overexpression induced caspase 3 gene expression and activity in melanomas [19]. Consistent with this, we found that caspase 3/7 activation was significantly increased in breast and lung cancer cells overexpressing RASSF1A which were treated with TNF-*α* compared to those overexpressing RASSF1C (Figure 4). We have previously shown that RASSF1C down-regulates caspase 3 expression and activity in breast cancer cells [8] and now in lung cancer cells as well. Thus our results show that RASSF1A and RASSF1C have opposite effects on caspase 3, both at the transcriptional and at the protein activation levels. This further supports a dual role of the RASSF1 gene.

Recently, RASSF1A has been demonstrated to interact with MST1/2 through the Salvador/Rassf/Hippo (SARAH) domain to activate apoptosis by relieving Raf1 inhibition of the MST1/2 signaling pathway, eventually leading to H2B phosphorylation and DNA fragmentation. Thus, we assessed MST1/2 phosphorylation in breast and lung cancer cells overexpressing RASSF1A or RASSF1C. We found that, while RASSF1A overexpression induced MST1/2 phosphorylation, RASSF1C overexpression attenuated MST1/2 phosphorylation in breast and lung cancer cells after TNF-*α* treatment (Figure 5). Also, overexpression of RASSF1C appears to reduce and overexpression of RASSF1A appears to increase the total MST protein levels (Figure 5(b)). This demonstrates another functional difference between RASSF1A and RASSF1C that may well affect apoptosis. Since both RASSF1A and RASSF1C contain the SARAH domain located in their identical C-termini, RASSF1C should be capable of interacting with the SARAH domain-containing proteins and could potentially attenuate MST1/2-mediated apoptosis. We have also assessed the phosphorylation status of the YAP protein, a downstream effector of the Hippo pathway. Breast and lung cancer cells overexpressing RASSF1A showed slightly higher levels of phosphorylated YAP compared to those cells overexpressing RASSF1C. Interestingly, levels of phosphorylated YAP were reduced in the same breast and lung cancer cells overexpressing RASSF1A or RASSF1C and in lung cancer cells overexpressing RASSF1C upon treatment with TNF-*α*. Since both RASSF1A and RASSF1C contain the SARAH domain located in their identical C-termini, RASSF1C should be capable of interacting with the SARAH domain-containing proteins and could potentially attenuate MST1/2-mediated apoptosis. More focused and detailed studies investigating the impact of RASSF1C on the Hippo and the MOAP1/Bax proapoptotic pathways are needed, and we are undertaking these studies.

**Figure 10:**
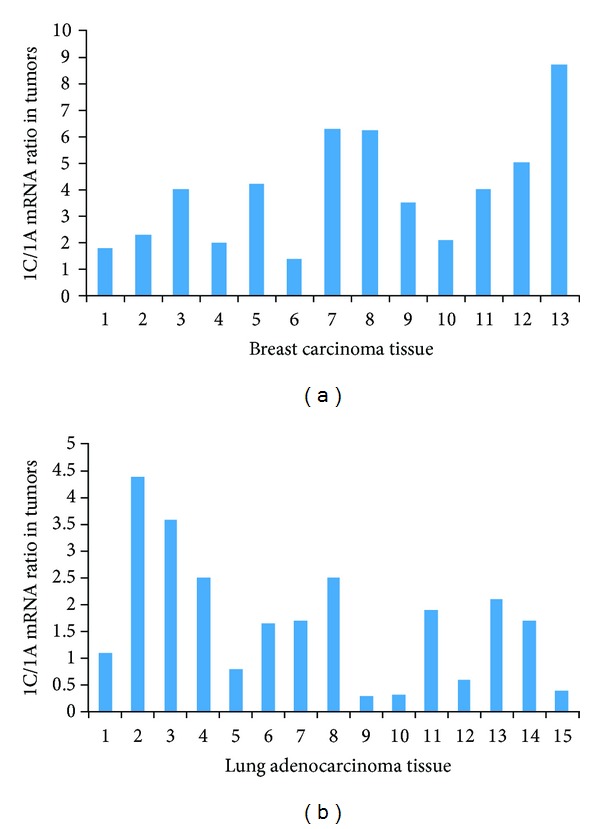
The ratio of RASSF1C to RASSF1A expression was assessed in breast and lung tumor tissues. The ratio of RASSF1C to RASSF1A was greater than 1 in 100% of breast (a) and 60% of lung (b) tumors. The ratio was calculated by dividing (RASSF1C expression in tumor tissue/RASSF1C in normal tissue) by (RASSF1A expression in tumor tissue/RASSF1A in normal tissue).

In this paper, we also assessed the expression profile of RASSF1A and RASSF1C in tumor and matched normal tissues using RT-PCR analysis. Published work demonstrates that RASSF1A is a tumor suppressor and its expression is downregulated in many different types of human cancers including breast and lung [1–5]. In contrast, RASSF1C is well expressed in most human solid tumors including breast and lung cancers. It has been suggested that the expression ratio of RASSF1A to RASSF1C may be critical when it comes to maintaining normal growth versus inducing oncogenic cell growth. Therefore, we assessed the mRNA levels of RASSF1A and RASSF1C in normal breast and lung tissues using RT-PCR. We found that RASSF1A mRNA levels are consistently and significantly higher than RASSF1C mRNA levels in all normal breast and lung tissues examined (Figure 7). It is possible that a higher RASSF1A/RASSF1C expression ratio would suppress the oncogenic growth effects of RASSF1C, and RASSF1A functions could be dominant. However, in cancers, the opposite may be true. When RASSF1A expression is downregulated due to methylation in cancer cells, the RASSF1C/RASSF1A expression ratio would allow for the growth-promoting and antiapoptotic actions of RASSF1C. Consistent with this, we found that the ratio of RASSF1C to RASSF1A is elevated in 100% of breast and 60% of lung tumors (Figure 10). Whether the increase of RASSF1C function is mainly due to a decreased RASSF1A expression alone or is due to a combination of suppression of RASSF1A expression and up-regulation of RASSF1C expression remains to be thoroughly investigated.

Previous studies have shown that RASSF1C expression is significantly upregulated and RASSF1A is significantly downregulated in lung and pancreatic tumor tissues and that up-regulation of RASSF1C correlates with poor prognosis and survival [11, 12]. Consistent with this, we also found that RASSF1A expression is significantly downregulated in 92% of breast tumors and 53% of lung tumors. On the other hand, RASSF1C was upregulated in 62% of breast tumors and 47% of lung tumors. Our findings show that RASSF1C expression is significantly elevated in half of breast and lung cancers, further supporting a growth promoting role for RASSF1C in these cancers. Obviously larger tissue sample sizes of breast cancer, lung cancer, and other cancer types of cancer will need to be examined.

## 5. Conclusions

Our findings suggest that RASSF1C has opposite effects on cell proliferation and apoptosis in breast and lung cancer cells compared to RASSF1A. Our current and previously published data suggest that RASSF1C can function as a growth promoting protein and further support the emerging concept of the dual functionality of the RASSF1 gene.
